# Editorial: Rising stars in avian physiology: 2024

**DOI:** 10.3389/fphys.2025.1691951

**Published:** 2025-09-05

**Authors:** Sandra G. Velleman, Colin G. Scanes

**Affiliations:** ^1^ The Ohio State University, Columbus, OH, United States; ^2^ University of Wisconsin-Milwaukee, Milwaukee, WI, United States

**Keywords:** broilers, early career, song birds, reproduction, thermal stress, satellite cells

In 2022, Frontiers in Avian Physiology published the initial Rising Stars Research Topic to aid in the development of researchers at early stages in their career with papers accepted from all disciplines in the area of Avian Physiology. The Research Topic was very successful with 46,508 article views and 16,272 article downloads. Thus, we viewed a Research Topic promoting those in the area of avian physiology to be critical to the sustainability of poultry and the poultry industry. As in the 2022 Rising Stars Research Topic, all areas of research in avian physiology were open for submission.

Bird reproduction was addressed by Singh et al., Bond et al., and Garcia-Meja et al. The timing and seasonal reproduction was studied by Singh et al. using dark-eyed Juncos. They found that gonadotropin releasing hormone was elevated in early breeding birds compared to migrating Juncos. With regard to commercial broilers, the presence of lipid biomarkers correlated with sperm mobility was reported on by Bond et al. The lipidome of the seminal plasma, sperm cell and whole semen was characterized in broilers with different sperm mobilities. Eight potential biomarkers of excellent sperm mobility were identified in this study. Although further evaluation is necessary, this may lead to screening strategies for fertility potential in roosters. On the hen side, Garcia-Meja et al. investigated physiological changes regulating calcium and phosphorus utilization which is essential for bone formation and eggshell mineralization. They found dietary supplementation with 1α-hydroxycholecalciferol increased medullary bone formation with increase bone mineral density in the humerus and tibia.

Birds are visual animals and have a complex system of vision. Bird vision was reported on by Seth et al., and Lingstӓdt et al. Seth et al. discussed how adeno-associated virus vectors are being used as gene therapy tools for the study and treatment of retinal disorders. These studies have been predominantly done using a murine model while retinal research in birds has been limited. In their novel study, adeno-associated virus serotypes were identified capable of transducing the avian retina. Lingstӓdt et al. reported on color perception in Jackdaws. They found that Jackdaws can discriminate colors based on their hue.

The effect of extrinsic stress on broilers was studied by Vaughn et al. and Harding et al. Extrinsic heat challenges from high ambient temperatures is a major factor affecting the poultry industry. Furthermore, the increase in temperature due to global warming is likely to augment the current problem further causing both financial and food production impacts. In the study by Vaughn et al., broiler chickens were exposed to an acute heat challenge on day 4 posthatch after a period of embryonic heat conditioning commencing on embryonic day 7 where temperature was increased to 39.5 °C for 12 h and then maintained at 37.5 °C for 12 h through embryonic day 16. They found that embryonic heat conditioning reduced the severity of heat stress on the hypothalamic response. Harding et al. reported on how various ventilation shutdown procedures affect laying hens where rapid depopulation is required in situations like highly pathogenic avian influenza. The three treatments evaluated were ventilation shutdown plus heat, ventilation shutdown plus heat and relative humidity, and ventilation shutdown plus carbon dioxide. Their results found that ventilation shutdown with heat and relative humidity is a good method for the industry to use to rapidly depopulate laying hen facilities.

Yu et al. and Powell et al. investigated the adult myoblast population of cells, satellite cells, in the breast muscle. Satellite cells are responsible for all posthatch muscle growth and the repair and regeneration of muscle. Traditionally, it was thought that satellite cells were a homogenous population of cells. Although varying rates of proliferation and differentiation have been found in satellite cells isolated from the same muscle fiber. Yu et al. using transcriptome analysis of satellite cells isolated from the same pectoralis major muscle with different proliferation rates found 5,300 genes differentially expressed. Thus, providing proof of functional diversity of satellite cells. Powell et al. reported on the control of satellite cell gene expression and regulation of the translation of mRNAs into proteins by non-coding circular RNAs in satellite cells isolated from 2 different lines of turkeys and the effect of temperature. Circular RNA expression was found to be significantly affected by thermal treatment and genetic background of the satellite cells with 140 differentially expressed circular RNAs.

Cherenetsov and Utvenko published a review article on how avian migrants know where they are going on their first migration. The question remains how birds who fly individually like songbirds, navigate their migration route when they are not with experienced conspecifics. Recent research has found that first time migrants follow geomagnetic cues and perhaps other cues to help them navigate their migration path.

